# The Role of CT-Angiography in the Acute Gastrointestinal Bleeding: A Pictorial Essay of Active and Obscure Findings

**DOI:** 10.3390/tomography8050198

**Published:** 2022-09-23

**Authors:** Marco Di Serafino, Francesca Iacobellis, Maria Laura Schillirò, Giuseppina Dell’Aversano Orabona, Alberto Martino, Raffaele Bennato, Antonio Borzelli, Gaspare Oliva, Chiara D’Errico, Filomena Pezzullo, Luigi Barbuto, Roberto Ronza, Gianluca Ponticiello, Fabio Corvino, Francesco Giurazza, Giovanni Lombardi, Raffaella Niola, Luigia Romano

**Affiliations:** 1 Department of General and Emergency Radiology, “Antonio Cardarelli” Hospital, Antonio Cardarelli St. 9, 80131 Naples, Italy; 2 Department of Gastroenterology and Digestive Endoscopy, “Antonio Cardarelli” Hospital, Antonio Cardarelli St. 9, 80131 Naples, Italy; 3 Department of Interventional Radiology, “Antonio Cardarelli” Hospital, Antonio Cardarelli St. 9, 80131 Naples, Italy

**Keywords:** gastrointestinal bleeding, haemorrhage, computed tomography angiography, dual-energy computed tomography angiography, vascular endothelial growth factor inhibitors

## Abstract

Gastrointestinal bleeding is a potentially life-threatening abdominal emergency that remains a common cause of hospitalisation. Although 80–85% of cases of gastrointestinal bleeding resolve spontaneously, it can result in massive haemorrhage and death. The presentation of gastrointestinal bleeding can range from asymptomatic or mildly ill patients requiring only conservative treatments to severely ill patients requiring immediate intervention. Identifying the source of the bleeding can be difficult due to the wide range of potential causes, the length of the gastrointestinal tract and the intermittent nature of the bleeding. The diagnostic and therapeutic approach is fully dependent on the nature of the bleeding and the patient’s haemodynamic status. Radiologists should be aware of the appropriate uses of computed tomography angiography and other imaging modalities in patients with acute gastrointestinal bleeding, as well as the semiotics of bleeding and diagnostic pitfalls in order to appropriately diagnose and manage these patients. The learning objective of this review is to illustrate the computed tomography angiography technique, including the potential role of dual-energy computed tomography angiography, also highlighting the tips and tricks to identify the most common and uncommon features of acute gastrointestinal bleeding and its obscure form.

## 1. Introduction

### 1.1. Definition and Incidence

Gastrointestinal bleeding (GIB) is a symptom of many digestive and/or systemic disorders or drug side effects, which can appear suddenly (i.e., acute forms) or can occur as a chronic/recurrent course.

Acute GIB is a potentially life-threatening abdominal emergency, with an estimated annual incidence of 100–200 cases per 100,000 population for the upper gastrointestinal tract and approximately 20–35 cases per 100,000 population for the lower gastrointestinal tract [1]. Although 80–85% of cases resolve spontaneously, GIB causes frequent hospitalisation and high morbidity and mortality, especially in the elderly and in patients with other comorbidities, with a higher incidence rate in men than in women (2:1) [1,2]. About 50% of hospital admissions for GIB relate to upper gastrointestinal bleeding (oesophagus, stomach and duodenum—upper gastrointestinal bleeding—UGIB), 40% relate to the lower gastrointestinal tract (colon and rectum—lower gastrointestinal bleeding—LGIB) and 10% is represented by haemorrhages of the middle tract (small intestine—small bowel bleeding—SBB or middle gastrointestinal bleeding—MGIB) (Table 1) [3].

### 1.2. Clinical Presentation

The clinical evaluation and appropriate triage of patients with acute gastrointestinal bleeding can be difficult. The manifestation of bleeding with haematemesis, coffee grounds vomiting, or the presence of a substantial amount of blood or coffee grounds-like material in the nasogastric tube aspirate indicate a proximal source of bleeding [4]. Melaena generally indicates a proximal source of bleeding but can occur with slight bleeding lesions of the small intestine or proximal colon [4]. Haematochezia generally indicates mild blood loss from the colon or anorectal area, whereas severe bleeding from the distal GI tract is defined as rectorrhagia. Maroon-coloured stools can be seen when the source of bleeding is located in the ileum or proximal colon [4]. In Table 1, the different presentations of GIB are classified.

### 1.3. Diagnostic Pathway

**Figure 1:**
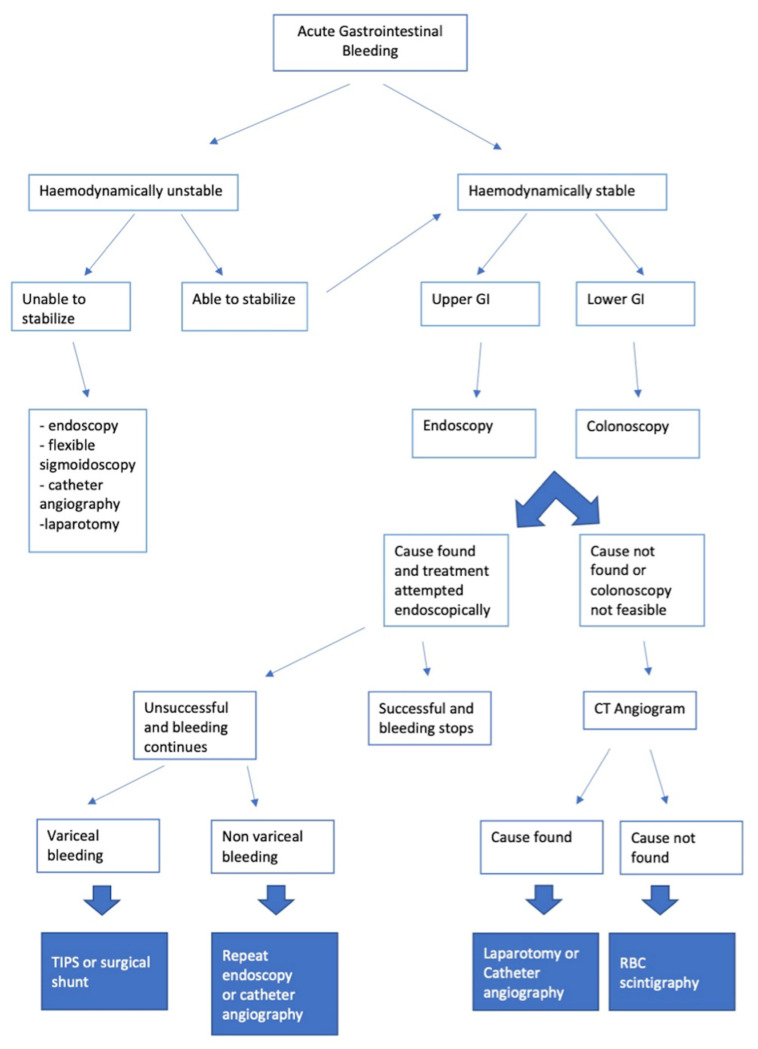
Therapeutic and diagnostic flow chart of a patient with gastrointestinal bleeding.

After the haemodynamic stabilisation of the patient, if required, finding the source of the bleeding in the upper or lower gastrointestinal tract is important as this affects the correct diagnostic pathway. In general, patients with suspected UGIB are triaged for upper GI endoscopy, while those with suspected LGIB and also patients with suspected SBB/MGIB (if the previous two causes are excluded) are more frequently evaluated with imaging or endoscopy of the lower GI tract depending on the various clinical scenarios (Figure 1) [2,3].

### 1.4. Diagnostic Endoscopic and Imaging Techniques

The goal of any diagnostic tool used in the context of a GIB event is to identify the source of bleeding early and, if possible, allow for prompt treatment of the haemorrhage. In many cases, GIB occurs intermittently or ceases spontaneously, constituting a diagnostic and therapeutic dilemma [5]. In Table 2, we propose a summary of the recommendations for the use of the different endoscopic and imaging techniques, highlighting their advantages and limitations.

Identifying the source of the bleeding can be difficult due to the wide range of potential causes, the length of the gastrointestinal tract and the intermittent nature of the bleeding [4,5,6,7,8,9,10,11,12]. The diagnostic and therapeutic approach is fully dependent on the nature of the bleeding and the patient’s haemodynamic status. The management of patients with GIB, therefore, requires a multidisciplinary approach that includes emergency physicians; internal medicine specialists; surgeons; gastroenterologists; diagnostic and interventional radiologists. Although many patients with GIB can be identified and treated without resorting to radiological imaging, the latter still plays an important role, especially in patients in whom endoscopy and/or medical management fail [12].

## 2. The Diagnostic Role of CTA

### 2.1. CTA: Appropriateness Criteria

The most recent guidelines on the appropriateness criteria drawn up by the American College of Radiology (ACR) for haemodynamically stable patients and especially those with LGIB, highlight a comparable diagnostic role in determining the bleeding site between scintigraphy and computed tomography angiography (CTA) [13]. However, in most hospital settings, CTA has become the first choice of radiological imaging for hemodynamically stable patients presenting with signs of GIB, especially in an emergency setting [12]. Indeed, CTA has a bleeding detection threshold (0.3–0.5 mL/min) similar to scintigraphy with tagged red blood cells (0.1–0.5 mL/min), which is usually not performed in an emergency setting, and superior to angiography (1.0 mL/min), which is usually performed only for therapeutic purposes [12,14]. CTA has the advantage of being able to accurately locate the source and the entity of arterial or venous gastrointestinal bleeding and identify the underlying pathology that may be causing the bleeding in order to guide subsequent management [2,3,4,5,12]. CTA is very accurate at identifying the bleeding site (close to 100%), providing information on vascular anatomy (anatomical variants, vessel occlusions, etc.) and indicating the need for a selective or super-selective angiographic procedure [12]. It is also considered a second-line procedure in patients with a previous endoscopy negative for GIB [12]. Intermittent bleeding represents a limitation for CTA, reducing the diagnostic sensitivity of the method from 91 to 92% in the diagnosis of the source of gastrointestinal bleeding in patients with active bleeding to 41–42% in those with obscure gastrointestinal bleeding; for the latter, follow-up or integration with other diagnostic methods becomes necessary. However, even when CTA cannot identify the exact site of the bleeding, it can still quickly recognise the lesion causing the intra- or extraluminal abnormalities or unexpected abdominal and/or thoracic disease [5,12].

### 2.2. Examination Technique

**Figure 2:**
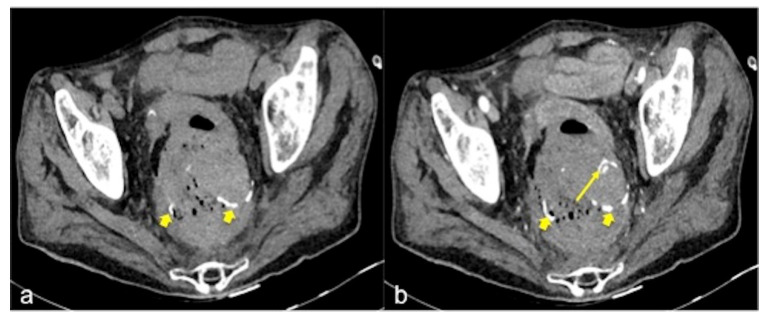
CT multiphasic study of haemorrhage at the surgical site of colorectal anastomosis. The pre-contrast CT axial image (**a**) shows spontaneous hyperdensity of metallic clips (small arrow). The arterial phase (**b**) shows another hyperdense spot (long arrow) near the anastomosis and is suggestive of active bleeding.

CTA should be performed in all patients using the multi-slice spiral technique, with cranio-caudal acquisition in the supine position with abducted upper limbs, to reduce the radiation dose and ensure greater image quality of the thoraco-abdominal organs. Acquisitions with the patient holding their breath allow for the avoidance of movement artefacts. The CTA protocol includes a preliminary low radiation dose scan of the abdomen and pelvis without an intravenous (IV) contrast medium. This is useful for recognising any pre-existing hyperdense material in the lumen of the bowel (e.g., suture material, clips, foreign bodies, orally administered drugs, coprolith, etc.) that can be mistaken for an active focal haemorrhage (Figure 2) [2,3,4,5,12,13,14].

Two or three phases are then obtained after IV administration of the contrast medium with coded scan times (Table 3): the arterial phase is obtained with the “bolus tracking” technique which allows the scan to be synchronised with the transit of the contrast medium in the aortic arch when the value of 100 Hounsfield units (HU) is reached and after having placed a region of interest (ROI) in this location; this scanning phase is extremely useful for documenting arterial extravasations of contrast medium and the vascular anatomy of the anatomical district examined [2,3,4,5,12,14]; then, with a delay of 70 to 90 s from the injection, the portal phase is obtained, which is useful for documenting the increase in arterial extravasation or haemorrhage of venous aetiology [2,3,4,5,12,13,14].

**Figure 3:**
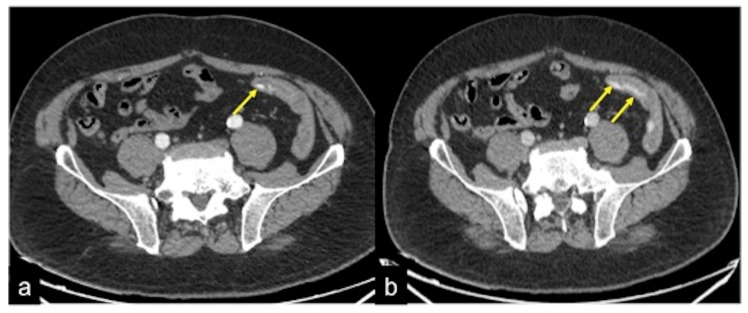
CT multiphasic study of bleeding at the proximal tract of sigma. In the arterial axial image after contrast media administration (**a**) it is possible to detect a blush of active bleeding (arrow). In the venous phase (**b**) a dimensional increase of the hyperdense blush is present (arrows) as a sign of active incremental bleeding in the venous phase.

**Figure 4:**
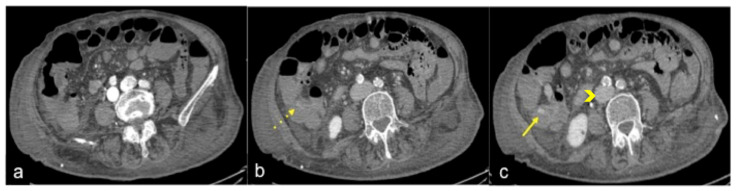
CTA multiphasic study of the haemorrhagic spot in the ascendant colon of a patient with heart failure. In the arterial phase of study (**a**), no signs of bleeding are evident. The venous phase (**b**) shows a hyperdense endoluminal blush of contrast media (discontinuous arrow) that is more evident in the successive phase ((**c**) arrow). CT multiphasic findings are suggestive of low-flow active bleeding in a patient with heart failure: note the inferior cava vein dilatation ((**c**) arrowheads).

In some cases, and especially in patients with reduced cardiac function, or with the finding of pathology in need of further investigation, a late venous phase 5 min from the start of the injection can also be considered (Figure 3 and Figure 4). For a successful scan, high concentrations of contrast medium are required (80–130 mL according to patient weight of 370–400 mgI/mL iodinated contrast medium), administered at high flow (3.5–4 mL/s) through a wide-gauge needle (18G cannula) into the antecubital vein; this is followed by a bolus of 40 mL of saline at the same flow rate to flush the previous IV administration of contrast medium [2,3,4,5,12,13,14]. The suggested acquisition volume includes the whole abdomen including the pelvis (i.e., from the diaphragm to the inferior pubic rami) and, in cases of presumed UGIB, such as oesophageal haemorrhage, it is recommended to also include the chest.

### 2.3. Post-Processing

**Figure 5:**
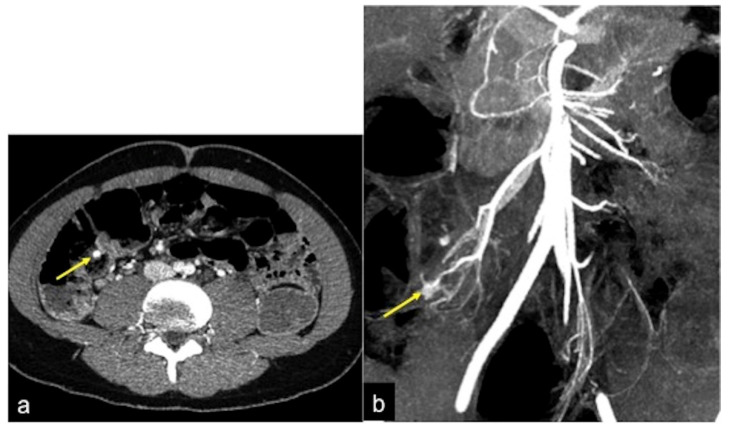
CTA study of a haemorrhagic spot in the ascendant colon. A vascular nidus (arrow) is detected in the arterial phase (**a**); the MIP post-processing with MPR coronal reconstruction facilitates the identification of the vascular malformation (arrow) in order to better define the vascular anatomy for the treatment approach (**b**).

**Figure 6:**
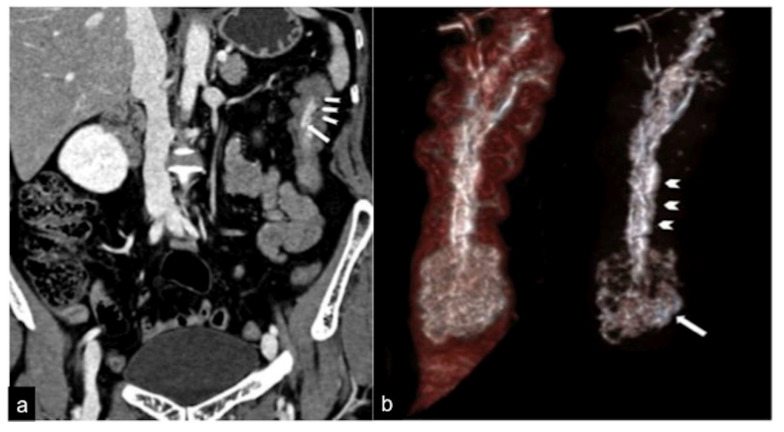
CTA coronal reconstruction in a patient with sub-occlusive recurrent status and occult haemorrhage. The coronal reconstruction (**a**) detects a pedunculated polyp (long arrow) with an arterial axis of vascular support. In the MIP post-processing with MPR and VR-3D, the coronal view (**b**) better defines the polypoid formation and the axis of vascular support (head arrows). Adopted with permission from ref. [15].

The images can be reconstructed at 5 mm thickness for those obtained without contrast medium (basal phase) and 1.25 mm thickness for those obtained in the arterial and venous (and late) phases [3]. The use of post-processing techniques, such as maximum intensity projection (MIP), multiplanar reformatting (MPR), curved multiplanar reformatting (cMPR) and 3D volume rendering image reconstructions, are extremely helpful in defining the anatomy and bleeding source (Table 3) [2,3,4,5,12,13,14]. The MPR and MIP images to be reconstructed also in the sagittal and coronal planes are useful, for example, to locate the bleeding intestinal segment and to map the artery affected by the haemorrhagic phenomenon in patients with an arterial bleeding source (Figure 5); they also provide information on vascular anatomy, since anatomical variants are frequent [5]. Virtual rendering-three dimension (VR-3D) images can also be useful for pre-operative details (Figure 6) [15]. In acute haemorrhage, a positive oral contrast agent could obscure contrast extravasation into the lumen while a neutral oral contrast agent can dilute contrast extravasation, making it more difficult to identify active bleeding; therefore, the use of oral contrast agents is not recommended in the event of active bleeding [3]. The goal of CTA is to identify blood in the bowel lumen, contrast extravasation and/or the cause of bleeding [3,12]. For occult GIB or suspected small bowel bleeding, because there is a slower bleeding rate, the goal of CTA is typically to identify the cause of the bleeding rather than subtle contrast extravasation; therefore, the study protocols may differ (Table 3) [3]. In general, the CTA protocols in use for GIB characterisation are balanced to maximise the sensitivity and specificity of the scan, while minimising the radiation dose [3,12]. Some centres use a single-phase CTA technique (e.g., enteric phase, portal venous phase with split bolus) to reduce the number of scans and consequently the radiation dose delivered, even if with this approach some arterial abnormalities may be missed [3]. One methodological possibility in haemodynamically stable patients with occult or intermittent GIB could be the use of CT enterography (CTE) with multiphase acquisition (Table 3) [3,12]. The main difference between a CTE and a CTA is that in CTE, the lumen of the small intestine is dilated with a neutral oral contrast medium (approximately 1.5–2.0 L). This distension of the lumen allows for optimal visualisation of the enhancement of the mucosa and of the wall of the small intestine after the administration of IV contrast medium, thus increasing the detection sensitivity of bowel wall lesions, whereas the administration of oral fluid may dilute the endoluminal blood eventually present. So this examination is not appropriate in the acute bleeding phase, but in absence of active bleeding to look for possible wall lesions. Although multiphase CTE protocols vary between different radiology institutes, the general principles of IV contrast medium administration remain the same as that already indicated for CTA [3]. CTE, therefore, has the potential to be integrated with endoscopic techniques and CTA itself in the search for occult bleeding sources (SBB/MGIB and LGIB) [3,12].

### 2.4. Dual-Energy CTA (DECTA)

**Figure 7:**
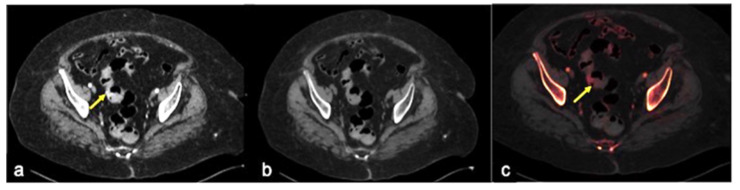
DECTA multiphasic study of a haemorrhagic spot in the middle sigmoid tract. In the arterial phase (**a**) an area of active bleeding is visible (arrow). In the virtual non-contrast post-processed axial image (**b**), it is possible to realise a selective subtraction of the contrast media density in order to obtain an image similar to the pre-contrast acquisition. In the iodinated distribution map of arterial phase (**c**), the blush of active bleeding is easily identified (arrow).

**Figure 8:**
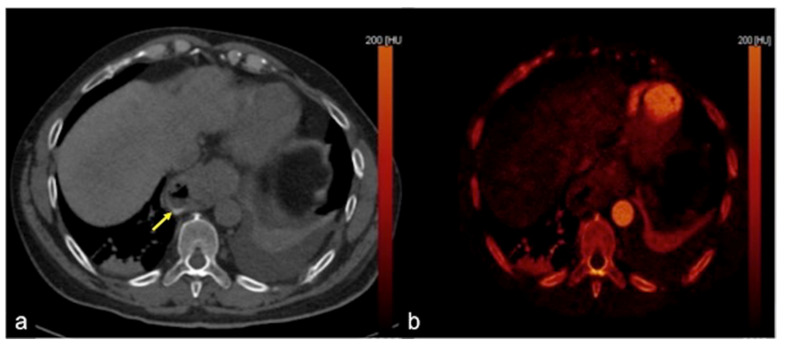
DECTA multiphasic study reconstruction. The virtual non-contrast axial image (**a**) shows an endoluminal linear hyperdensity in the distal oesophagus (arrow). In the iodinated map axial image (**b**) the hyperdensity is not magnified. These findings are suggestive of the presence of a foreign body, confirming the high confidence of DECTA post-processing diagnostic performance.

**Figure 9:**
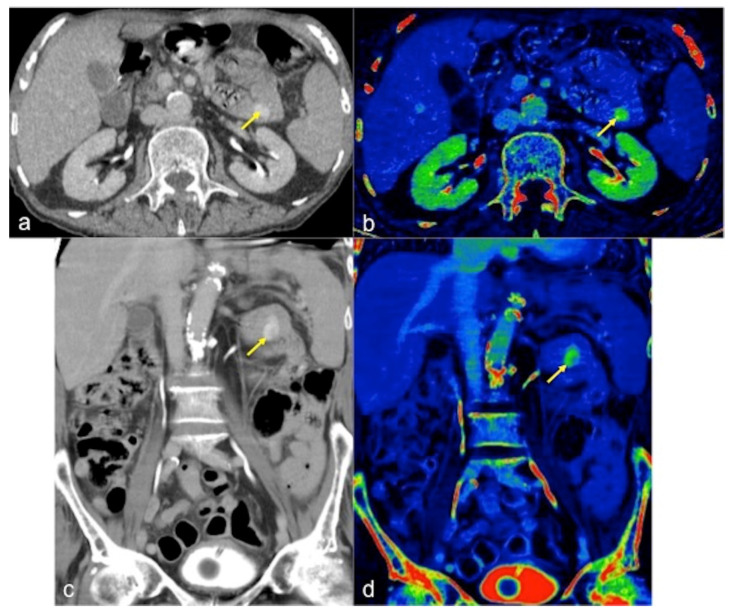
DECTA multiphasic study of active bleeding in the proximal jejunum. Thanks to axial (**a**,**b**) and coronal (**c**,**d**) images, it is possible to easily compare the traditional greyscale and the colourimetric maps of iodium distribution; a haemorrhagic focus of active bleeding is detected with high sensitivity ((**a**–**d**) arrow).

Technological advances in the field of CT have made it possible to develop post-processing techniques exploiting dual-energy technology (dual-energy CTA—DECTA), which offers slight advantages over “traditional” CTA (and CTE) by scanning the same structures anatomical with different kilovoltages (lower energy at 80 kV or 100 kV and higher energy at 140 kV) [10]. DECTA, by combining the information received from two different kilovoltages and comparing it to known densities such as those of water and iodine, allows for improvement of the contrast resolution through the use of lower doses of IV contrast medium (about 50% less than a conventional CT). Virtual pre-contrast scans are obtained by subtraction with an overall reduction in the radiation dose applied to the patient (approximately 30% less than conventional CT) (Figure 7 and Figure 8) [16,17]. Furthermore, through the analysis of the images obtained at low kiloelectron voltage (virtual monoenergetic) and also through the analysis of the iodine and chromatic distribution maps, the ability to define and diagnose a focal active haemorrhage is indeed facilitated (Figure 9) [3,16,17]. This is particularly important in detecting an active focal bleed, especially in the context of a conspicuous haemorrhage or ingested hyperdensity that stretches the lumen. In addition, metal density suppression techniques can be important to reduce artefacts from hardening of the radiant beam due to surgical clips, implants, foreign bodies etc., and improve the possibility of detecting extravasated contrast medium in the vessel lumen, identifying it as a bleeding point [3].

### 2.5. Semiotics of Bleeding and Diagnostic Pitfalls of CTA

**Figure 10:**
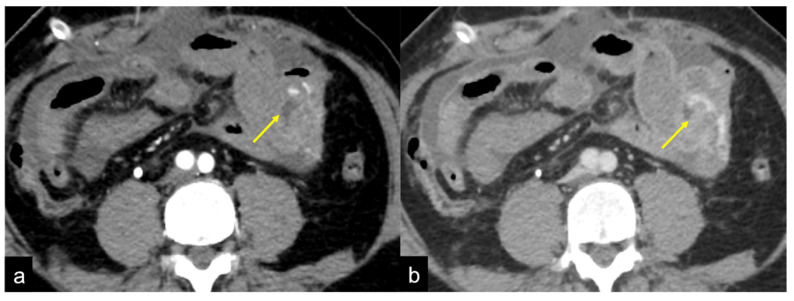
CT multiphasic study of active bleeding in the jejunum. In the arterial phase (**a**), a blush of active bleeding is detected (arrow). In the venous phase (**b**), the increase in haemorrhagic blush is documented with contiguous small bowel loop involvement (arrow).

The critical sign of haemorrhage detected by CTA imaging is a “blush” of contrast medium in the lumen of an intestinal loop, detectable as a focus of high attenuation (about 90 HU) not present in the images acquired in the basal phase or in the dual-energy virtual reconstructions; in the venous phase, this also tends to change in size and morphology assuming a lower position in the lumen of the intestinal loop or it can be moved by peristalsis, taking on a more irregular shape (Figure 3 and Figure 10) [2].

**Figure 11:**
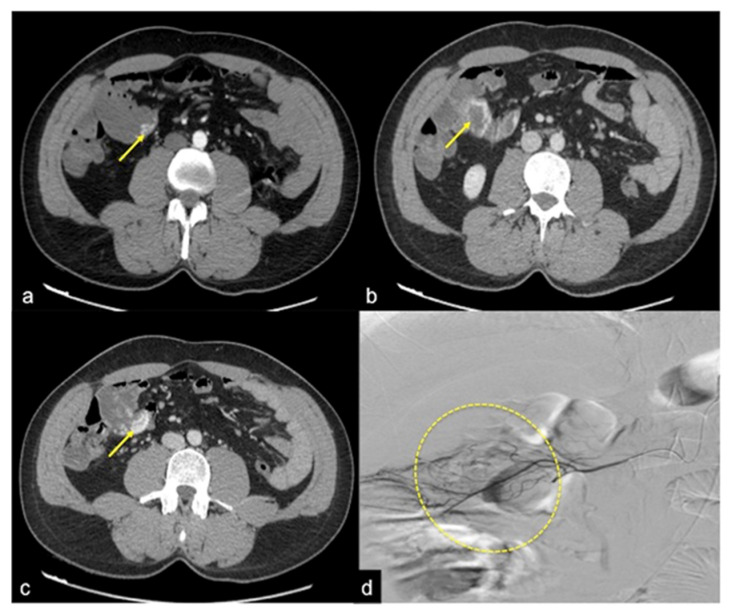
CTA multiphasic study of small intestine angiodysplasia. In the arterial phase (**a**) an active blush of contrast media is detected at the small bowel wall (arrow). The venous (**b**) and late (**c**) phases show a gradual increase of contrast blush (arrow (**b**)) with consequent production of a haematic collection (arrow (**c**)). In the selective angiography (**d**) the CTA findings are confirmed and the presence of an angiodyslastic focus is documented (discontinued circle).

Beyond the detectable attenuation values of the haemorrhagic focus in the bowel loop lumen, which, however, may not always reach the critical threshold of 90 HU, especially if thin or diluted, the best approach for the identification and diagnosis of GIB consists in combining the findings from the arterial phase to those of the basal and venous phases; it is, precisely, the changing inter-phasic appearance (from arterial to venous) of the extravasated contrast medium that unequivocally confirms the presence of bleeding, especially when, in the preliminary basal phase, no hyper-attenuating material is detected in the region of the lumen (Figure 11) [18].

**Figure 12:**
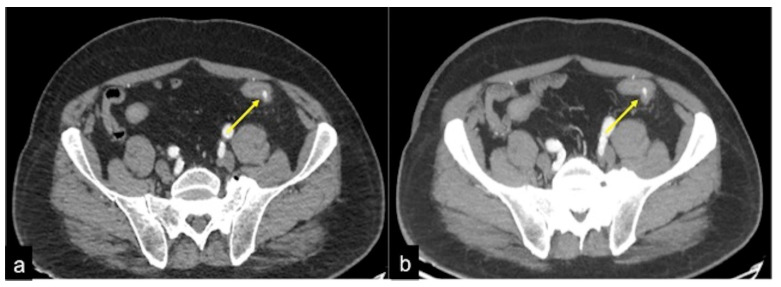
CTA arterial phase (**a**) and relative MIP reconstruction (**b**) of a haemorrhagic spot (**a**,**b**, arrow) in the descending colon.

**Figure 13:**
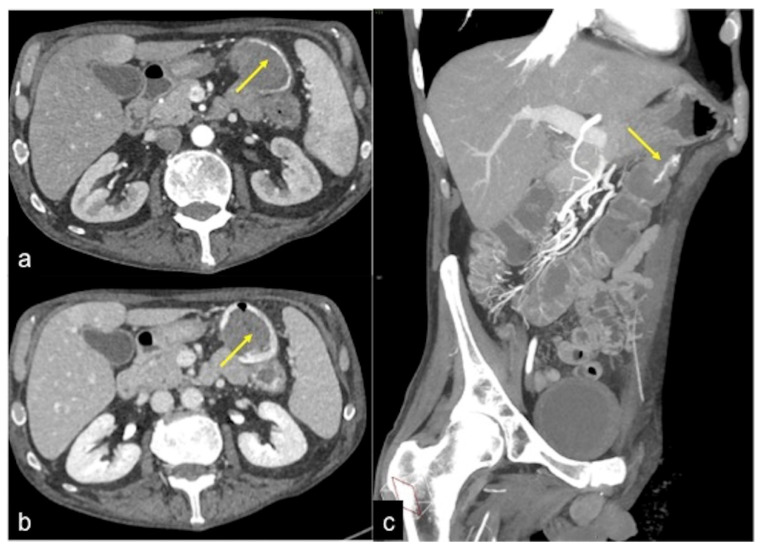
Different radiologic pathways of bleeding. CTA multiphasic study in arterial (**a**) and venous (**b**) phases reveals intestinal bleeding with circular morphology (**a**,**b**, arrow) along the endoluminal wall of splenic flexure with enlargement and intensification in the venous phase. The MPR reconstruction of the arterial phase in the coronal-oblique plane (**c**) shows the whorled morphology of bleeding (arrow).

**Figure 14:**
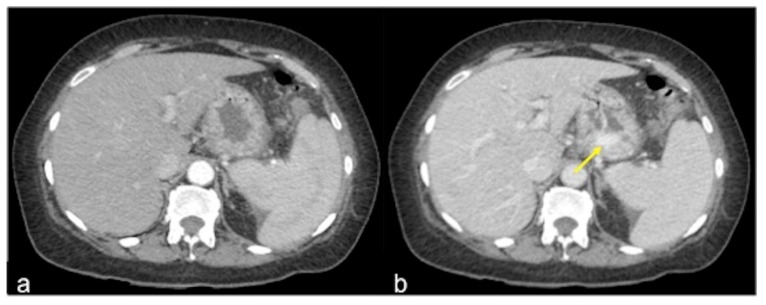
CTA multiphasic study of venous gastric bleeding in arterial (**a**) and venous (**b**) phases. In the venous phase, it is possible to see an amorphous haemorrhagic collection ((**b**) arrow), not detectable in the arterial phase.

The extravasation of the contrast medium itself can have a variety of morphologies and appearances according to the extent and entity of the extravasation, the upper or lower location of the loop wall from which it originates, but also the underlying cause. It commonly assumes morphologies which can be linear and jet-like, swirling, circular or ellipsoidal, clustered or cloud-shaped, or it may even form a fluid-contrast level (Figure 12, Figure 13 and Figure 14) [2,18].

**Figure 15:**
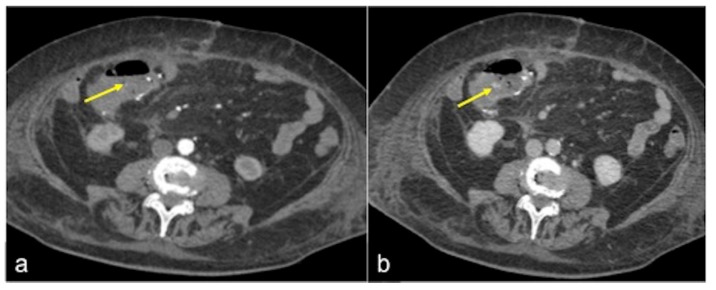
CTA multiphasic study of slow flow bleeding in arterial (**a**) and venous (**b**) phases. The haemorrhagic spot at the surgical site of ileo-colic anastomosis appears inconspicuous in the arterial phase ((**a**) arrow) and is better identifiable in the venous phase ((**b**) arrow).

**Figure 16:**
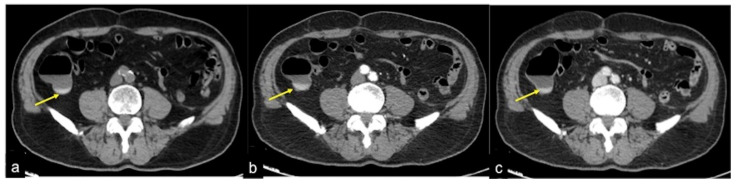
CTA multiphasic study of caecum sentinel clot in a patient with hematochezia. A small amount of spontaneous hyperdensity is collected at caecal fondus, resulting visible in the pre-contrast (**a**), arterial (**b**) and venous (**c**) phases with analogous morphologic and densitometric characteristics (**a**–**c**, arrow). This finding is suggestive of a sentinel clot and presumable recent bleeding.

Furthermore, it should be considered that in patients with low-flow bleeding, contrast extravasation of arterial origin may be detectable in the venous phase, while in patients with recent bleeding but who are not bleeding at the time of CTA, only a hyperdense clot is found within the intestinal lumen, defined as a “sentinel” clot or indicator of the site of previous active bleeding (Figure 4, Figure 15 and Figure 16) [2,3,18].

**Figure 17:**
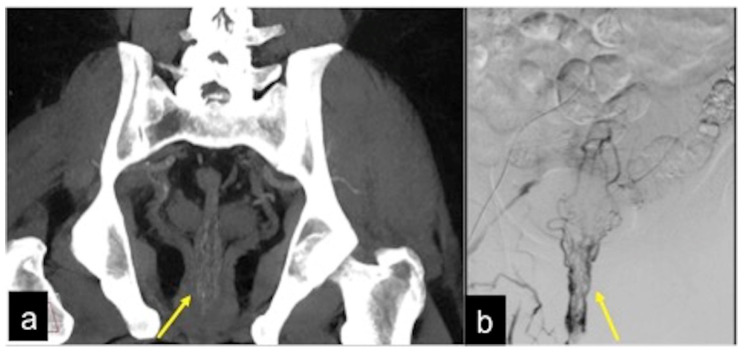
MIP reconstruction of arterial phase (**a**) and angiography study (**b**) of rectal artero-venous malformation. The anatomic detail is very similar when comparing imaging of the two techniques ((**a**,**b**) arrow).

**Figure 18:**
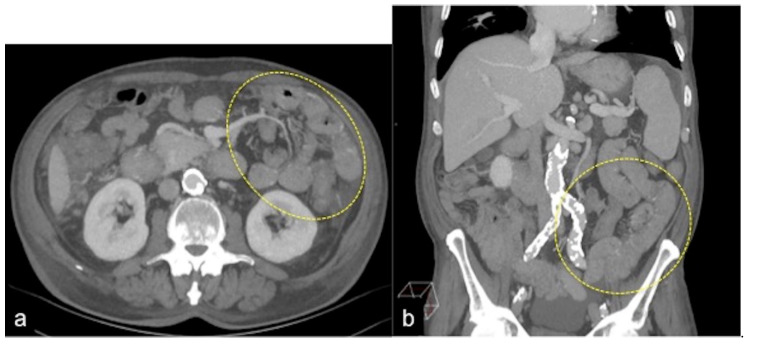
Axial (**a**) and coronal (**b**) MIP reconstruction of the arterial phase in a patient of occult gastrointestinal bleeding. The MIP post-processing permits the intensification of the density of thin vascular structures in the wall (**a**,**b**, discontinuous circle) of the jejunum and proximal ileus as signs of small bowel telangectasia.

In all cases of GIB, post-processing appears to be fundamental, i.e., carefully examining the MIP reconstructions, because they can identify unseen subtle bleeding in non-reconstructed images or better highlight vascular malformations with an anatomy similar to that of an angiographic study (Figure 5, Figure 17 and Figure 18) [2,3,18].

It also appears important to be well aware of the common diagnostic pitfalls in interpreting CTA in the context of GIB (Table 4) [19].

**Figure 19:**
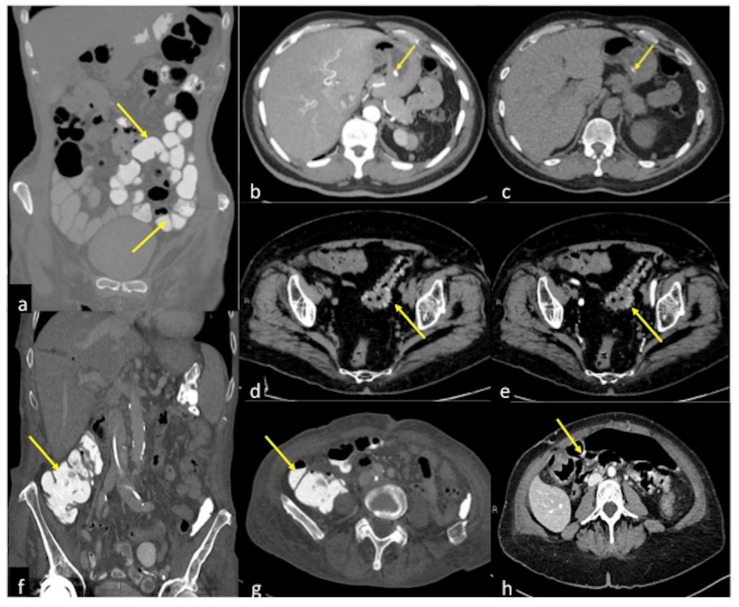
Pitfalls in the detection of acute gastrointestinal bleeding. (**a**) Positive contrast media administration (arrow): coronal CTA reconstruction of contrast media distended bowel loops (arrows) obscure any GIB source. (**b**,**c**) Endoluminal hyperdense ingested capsule: arterial and pre-contrast axial CTA image of an endoluminal hyperdense ingested capsule in patients with melaena. In the arterial phase with MIP post-processing (**b**), an endoluminal hyperdense inclusion is detected (arrow) in the gastric lumen; the pre-contrast phase (**c**) confirms the presence of the inclusion with analogous morphologic and densitometric characteristics (arrow). (**d**,**e**) Retention of contrast media in patients with diverticulosis and haematochezia: pre-contrast (**d**) and arterial (**e**) axial CTA images show unchanged hyperdense diverticula (**d**,**e**, arrow). (**f**,**g**) Hyperdense fecaloma: coronal (**f**) and axial (**g**) CTA image of hyperdense fecaloma (**f**,**g**, arrow) due to retained contrast media. (**h**) Cone-beam artefacts: axial arterial CTA image shows massive hyperdensities within the bowel lumen (arrow) due to cone-beam artefacts.

For example, fluid distension of the bowel loops can dilute the contrast extravasation, potentially causing a false negative result. High-density material in or near the bowel lumen, including surgical clips, ingested material and faecaliths, can be mistaken for haemorrhage without first viewing the basal or virtual phase without contrast (Figure 19) [2,3,18].

Cone-beam artefacts can also lead to the false appearance of high-density content within the bowel lumen although post-processing techniques in DECTA can at least partially overcome these limitations [3].

**Figure 20:**
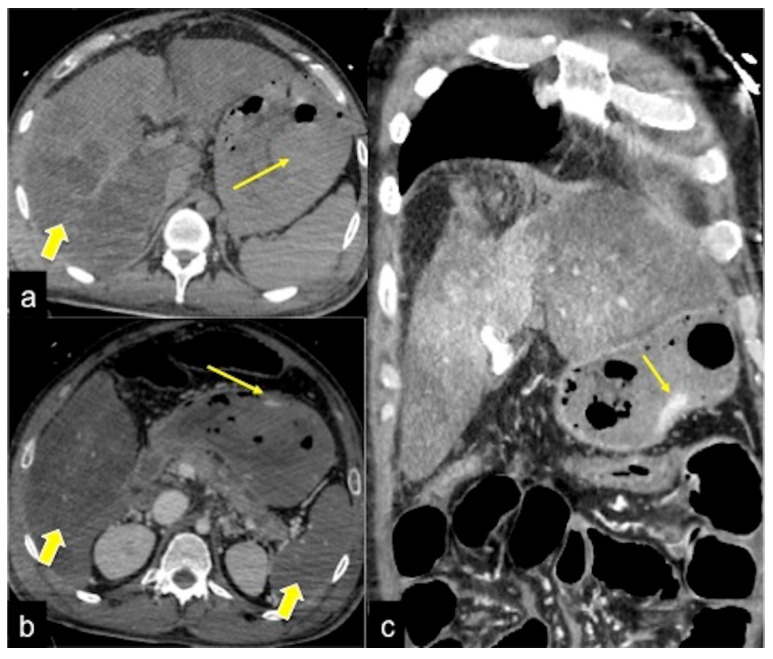
CTA multiphasic study of gastric bleeding in hypopefusive complex. The pre-contrast phase (**a**) shows a gastric endoluminal area of spontaneous hyperdensity (long arrow) as a sentinel clot associated with alteration of liver density in the right lobe (large arrow). In the arterial phase (**b**) an active haemorrhagic focus in the stomach (long arrow) associated with hypo perfusion of the liver and splenic parenchyma (large arrows) are detected as signs of hypotensive general status. In the venous phase (**c**) there is an enlargement of the haemorrhagic blush (long arrow) with inhomogeneous liver perfusion.

Not forgetting the countless collateral findings that may be encountered during the CTA examination, a separate mention should be made of the early signs indicative of hypovolaemic shock in severe bleeding states, which fall under the umbrella term of “CT hypoperfusion complex” (Figure 20) [20].

## 3. Upper Gastrointestinal Bleeding (UGIB)

UGIB can arise from pathological conditions of the oesophagus, stomach or duodenum and is an important emergency condition. The aetiology is traditionally divided into variceal and non-variceal causes; the latter is subdivided into non-vascular and vascular causes, including, among others: peptic ulcers, 28–59% (duodenal ulcer 17–37% and gastric ulcer 11–24%); erosive disease of the oesophageal/gastric/duodenal mucosa, 1–47%; Mallory–Weiss syndrome, 4–7%; malignant tumours of the upper GI tract, 2–4%; other diagnoses, 2–7%; no precise cause identified, 7–25% [4,21]. In particular, gastrointestinal bleeding related to the increasing consumption of non-steroidal anti-inflammatory drugs (NSAIDs) and antiplatelet agents is on the rise, especially in the elderly [4,21]. According to the appropriateness criteria of the American College of Radiology (ACR) for UGIB, endoscopy of the upper digestive tract is the investigation of choice for diagnosis and therapy while, usually, radiological diagnostic and therapeutic management does not play a significant role in this context [12,22]. However, there are four situations/variants, according to the ACR appropriateness criteria, in which radiological management is potentially useful: (I) endoscopy reveals a non-variceal arterial bleeding source: angiography and CTA are equally useful. If the patient is hemodynamically unstable, angiography is the gold standard. CTA may not be diagnostic if the bleeding is intermittent; (II) endoscopy reveals non-variceal haemorrhages but does not identify a clear source: angiography and CTA are equally useful; (III) endoscopy is negative: includes obscure UGIB. Angiography and CTA are comparable but can have false negatives. Angiography has the highest diagnostic yield, but can be limited by arterial anatomical variants; (IV) endoscopy is contraindicated: angiography and CTA are comparable in these patients. Table 5 summarises the most frequent causes of UGIB with the CT findings.

**Table 5:** Frequent causes of UGIB: clinical presentation and CT findings.

**UGIB DUE TO VARICES**		
	**Clinical Presentation**	**CT Findings**
**Oesophageal-gastric varices due to portal hypertension** (Figure 21)	Asymptomatic until they rupture in the oesophageal lumen and cause haematemesis or melaena or haematochezia depending on the severity of the bleeding.	Tortuous, enlarged, smooth tubular structures protruding into the oesophageal lumen or adjacent to the internal oesophageal mucosa.
**NON-VARICEAL NON-VASCULAR CAUSES OF UGIB**		
	**Clinical Presentation**	**CT Findings**
**Mallory–Weiss Tear** (Figure 22)	A history of recent retching or haematemesis or “coffee grounds” emesis following violent vomiting, often after excessive alcohol consumption, and manifested by stabbing pain in the epigastrium and left side of the chest, radiating to the back.	Finding haemorrhagic spots or foci of extraluminal gas at the site of the mucosal laceration.
**Oesophagitis** (Figure 23)	Anaemia, retrosternal pain.	Diffuse oesophageal thickening, submucosal oedema and mucosal hyperaemia.
**Oesophageal ulcer**	Haematemesis, epigastric pain and odynophagia.	Thickening of the wall, peri-oesophageal gas and fluid collection, extraluminal contrast extravasation.
**Oesophageal diverticulum** (Figure 24)	Asymptomatic bleeding.	Haemorrhage in a focal herniation of the mucosa through a site of weakness in the muscle layer.
**Peptic ulcer** (Figure 25)	Manifestations range from asymptomatic to melaena or haematemesis, to hypovolaemic shock. Bleeding due to gastric ulcers often presents with haematemesis, while duodenal bleeding can present with tarry stools or even occasionally haematochezia, depending on the extent of bleeding.	Direct identification of active haemorrhage as extravasation of an intra-luminal “jet” or “blush” of contrast medium at the site of the haemorrhage, detected in the gastric fundus or duodenal lumen.
**Neoplasia**	Anaemia in a patient with a history of cancer.	A focal area of high attenuation within the bowel lumen that represents a bleeding point at the tumour site.
**Gastrointestinal Stromal Tumour (GIST)** (Figure 26)	Asymptomatic or bleeding.	Soft tissue density mass with variable areas of necrosis. They are usually highly vascularised and the enhancement of the lesion may vary from homogeneous to peripheral and irregular depending on the lesion dimension and grade of malignancy.
**NON-VARICEAL VASCULAR CAUSES OF UGIB**		
	**Clinical Presentation**	**CT Findings**
**Dieulafoy Lesion** (Figure 27)	Melaena, haematemesis, haematochezia, or a combination of more than one of these signs, depending on the location of the lesion.	Abnormally enlarged submucosal vessel, which may appear tortuous, linear or as a non-specific “blush” of contrast medium at the mucosal/submucosal level.
**Artero-Venous Malformations**	Asymptomatic.	A tiny nidus of vascular potentiation appreciable in the arterial phase and often undetectable in the late phase. When associated with an early draining vein in the arterial phase, the lesion represents an AVM.
**Aorto-Gastric Fistula** (Figure 28)	Copious bleeding.	A connection between the aorta and the gastric lumen. Absence of adipose cleavage planes.
[23,24,25]		

## 4. Middle Gastrointestinal Bleeding/Small Bowel Bleeding (MGIB/SBB)

SBB should be suspected in patients with GIB and negative upper and lower endoscopy [3,4]. It is less common than UGIB and LGIB, accounting for only about 5–10% of gastrointestinal bleeds [4]. Overt small bowel bleeding refers to patients presenting with melaena or haematochezia with an identified bleeding site in the small intestine. Currently, the term obscure gastrointestinal bleeding (OGIB) which largely includes this category of bleeding should be reserved for patients with GIB and negative small bowel examinations, including video capsule endoscopy (VDE), endoscopy, enteroscopy and radiographic examinations [3,4]. Due to the relatively technically difficult endoscopic exploration of the small intestine and the limited availability of VDE and/or enteroscopy in non-reference centres, the small intestine may represent a blind zone and patients with SBB usually undergo multiple diagnostic investigations, hospitalisations and blood transfusions. The most common lesions responsible for SBB are vascular; other causes are tumours, inflammatory lesions and drugs, as well as rare causes such as haemobilia, haemosuccus pancreaticus and aorto-enteric fistula [4]. Vascular lesions and lesions of the small intestine induced by non-steroidal anti-inflammatory drugs (NSAIDs) are common causes of gastrointestinal bleeding in the small intestine in the elderly, while tumours, Meckel’s diverticulum, Dieulafoy’s lesion, and Crohn’s disease are common causes in patients under 40 years of age [4]. According to the ACR appropriateness criteria for SBB/MGIB, CTA is comparable to video capsule and superior to scintigraphy [13]. Table 6 summarises the most frequent causes of SBB/MGIB with the CT findings.

**Table 6:** Frequent vascular and non-vascular causes of SBB/MGIB: clinical presentation and CT findings.

**NON-VASCULAR CAUSES**		
	**Clinical Presentation**	**CT Findings**
**Tumour** (Figure 29)	Asymptomatic or bleeding.	Irregular wall thickening with foci of active bleeding.
**GIST**	Asymptomatic or bleeding.	Soft tissue density mass with variable areas of necrosis. They are usually highly vascularised and the enhancement of the lesion may vary from homogeneous to peripheral and irregular depending on the lesion dimension and grade of malignancy.
**Ulcer**	Obscure bleeding.	Thickening of the walls and fluid collections, extravasation of contrast medium. CT is poorly sensitive in the detection of superficial lesions.
**Meckel’s Diverticulum** (Figure 30)	Asymptomatic or, rarely, massive gastrointestinal bleeding.	A diverticulum with fluid or air content originating from the antimesenteric side of the distal ileum.
**Jejunal-Ileal Diverticulum**	Asymptomatic or, rarely, massive gastrointestinal bleeding.	Similar to Meckel’s diverticulum.
**Aorto-Enteric Fistula** (Figure 31)	Bleeding in a patient with a history of surgery for aortic aneurysm.	A connection between the aorta and the intestinal lumen. Absence of adipose cleavage planes.
**Haemobilia** (Figure 32)	Melaena, haematemesis, biliary colic, jaundice, or massive bleeding in a patient with a history of blunt or iatrogenic abdominal trauma.	Presence of blood in the gallbladder and biliary tree.
**Pancreatic Haemorrhage**	Intermittent epigastric pain in the abdomen, gastrointestinal bleeding (melaena, haematemesis, haematochezia) and raised serum amylase.	Pseudoaneurysm or pseudocyst with signs of active bleeding, associated with the finding of hyperdense material in the pancreatic ducts.
**VASCULAR CAUSES**		
**Angiodysplasia** (Figure 11)	Obscure bleeding.	Abnormally dilated, tortuous, thin-walled vessels involving small capillaries, veins and arteries.
**Telangiectasia** (Figure 18)	Iron deficiency anaemia with recurrent gastrointestinal bleeding.	Punctate area of enhancement with direct connections between arteries and veins.
**Dieulafoy’s Lesion**	**Obscure bleeding.**	Abnormal arteries typically protruding through a small mucosal defect ranging in size from 2 to 5 mm.
**Venous Lesion**	Obscure bleeding.	Varices may be visible in the enteric phase and become more intense in the late phase, with progressive filling of the mesenteric-systemic collateral veins.
**Venous Angioma**	Obscure bleeding.	Globular enhancement. Sometimes, phleboliths within the lesions, which are more visible in the arterial phase.
[4]		

## 5. Lower Gastrointestinal Bleeding (LGIB)

The term LGIB refers to bleeding from the colon and anus. Acute lower digestive bleeding accounts for approximately 20% of all major gastrointestinal haemorrhages [4,26,27,28,29,30]. Although in about 80% of cases it ceases spontaneously, bleeding can become a genuine gastroenterological emergency, with the need for hospitalisation and specialist assessment [4]. The most common causes of colonic bleeding are diverticulosis, haemorrhoids, ischaemic colitis, angioectasia and carcinoma (more common in the elderly); inflammatory bowel disease or polyps (more common in adolescents or young adults); other less common causes are post-polypectomy haemorrhage, infectious colitis, stercoral ulceration, colorectal varices, radiation proctopathy, NSAIDs-induced colopathy and Dieulafoy’s lesion [4]. In most patients with rectorrhagia and haematochezia, the bleeding originates from the colon. As such, the first-choice endoscopic examination is colonoscopy. Patients with LGIB must first be resuscitated with medical measures. When stabilised, they should undergo colonoscopy after preparation with polyethylene glycol [4]. According to the ACR appropriateness criteria for LGIB, there are four scenarios/variants [13]: (I) patients with active bleeding who are haemodynamically stable: colonoscopy is the most appropriate initial investigation method. In terms of radiological imaging, CTA and scintigraphy are equally useful, but CTA has many advantages over scintigraphy; (II) patients with active bleeding who are haemodynamically unstable: angiography is the most appropriate initial imaging method; (III) post-colonoscopy treatment with haemorrhage/continuous haemorrhage due to LGIB: angiography is the most appropriate imaging method; Table 7 summarises the most frequent causes of LGIB with the CT findings.

**Table 7:** Frequent vascular and non-vascular causes of LGIB: clinical presentation and CT findings.

	**Clinical Presentation**	**CT Findings**
**Diverticulosis** (Figure 33)	Asymptomatic or bleeding.	Protruding sacs where the vessels pass through the muscularis layer, between the mesenteric and antimesenteric taenia.
**Angiodysplasia** (Figure 5 and Figure 34)	Asymptomatic or bleeding.	Small hyperdense nodules within the intestinal wall, best defined in the portal phase of the study.
**Arterio-venous Malformation** (Figure 17)	Haematochezia-rectorrhagia.	Vascular nidus with early opacification of the veins in the arterial phase.
**Dieulafoy’s Lesion**	Asymptomatic or bleeding.	Abnormally enlarged submucosal vessel, which may appear tortuous, linear or as a non-specific “blush” of contrast medium at the mucosal/submucosal level.
**Rectal Varices and Haemorrhoids** (Figure 35)	Pain and/or bleeding.	Dilated veins with possible bleeding visible in the portal phase; rectal varices are located proximal to the linea dentata while haemorrhoids are located in the anus.
**Colorectal Cancer/Polyps** (Figure 6, Figure 36, Figure 37 and Figure 38)	Bowel obstruction with or without bleeding.	Adenocarcinoma: irregular wall thickening with or without stenosis [25]; Polyps: mass-forming protrusions in the intestinal lumen with vascularised peduncle.
**Inflammatory Bowel Disease** (Figure 39 and Figure 40)	Haematochezia-rectorrhagia.	Acute: thickening of the walls, engorgement of the adjacent vasa recta, hyperaemia of the mucosa and infiltration of perirectal fat. Chronic: the colon and rectum are narrowed and shortened, without haustra, and with proliferation of the perirectal fat.
**Colitis** (Figure 41)	It depends on the aetiology.	Non-specific but associated with medical history, the clinical history and location of the lesions, it may be useful for diagnostic purposes.
[26,30]		

## 6. Gastrointestinal Bleeding in Patients Treated with Vascular Endothelial Growth Factor (VEGF) Inhibitors

Drugs that can lead to GIB are well known and they include NSAIDs such as diclofenac and ibuprofen, platelet inhibitors such as acetylsalicylic acid, clopidogrel and prasugrel, as well as anticoagulants such as vitamin-K antagonists, heparin or direct oral anticoagulants [31]. In addition, it seems worthy to consider as an emerging problem the GIB side effects of anti-angiogenic drugs [27]. Anti-angiogenic drugs, especially anti-vascular endothelial growth factor (VEGF) agents, have entered the clinical armamentarium against cancer [32].

Angiogenesis plays a vital role in tumour growth and the spread of metastases. During the past decade, the inhibition of angiogenesis has been a focus of cancer therapies [27].

VEGF is a survival factor for endothelial cells and produces the abnormal phenotype of blood vessels within tumours. The use of VEGF inhibitors (VEGF-antibodies) is based on the assumption that the vascularity of the tumour can be destroyed while sparing other non-malignant vessels.

**Figure 42:**
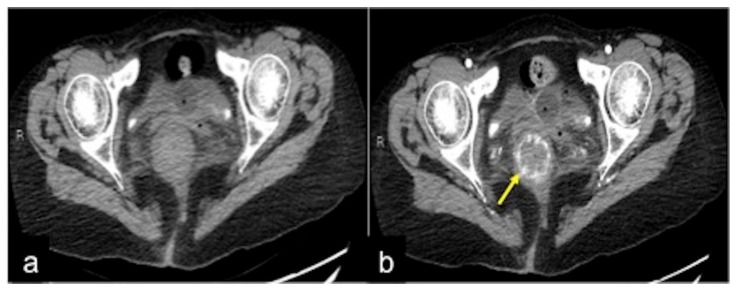
CTA multiphasic study of rectal bleeding during treatment with VEGF inhibitors. The pre-contrast (**a**) and arterial (**b**) phases show the presence of bleeding in the rectal wall (**b**, arrow) of a patient treated with Bevacizumab.

The increasing use of VEGF inhibitors has also resulted in a better understanding of their mechanism of action, increasing confidence in the main adverse effects associated with their long-term use [33]. Based on the influence of VEGF on the survival, stability and function of vascular tissue [34], it also remains an important factor for the architecture and integrity of the microvasculature in normal tissues. For this reason, when VEGF signalling is blocked, the ability of endothelial cells to repair and renew in response to any stimulus may be impaired, resulting in an increased risk of bleeding [29]. Ongoing gastrointestinal bleeding associated with Bevacizumab is a common side effect observed in clinical trials with life-threatening consequences [34,35]. The most common site of high-grade bleeding associated with Bevacizumab is the rectal tract in patients treated for any type of cancer (Figure 42) [33,34,35]. Tumour necrosis is a major bleeding risk factor. In fact, the proliferation of neoplastic cells and the invasion of the hollow organ walls are the main behavioural characteristics of gastrointestinal tumours that contribute to intestinal tumour necrosis. Another important risk factor for ongoing gastrointestinal bleeding is the presence of unknown gastroduodenal ulcers, submucosal telangectasias, submucosal varices, and pseudoaneurysms. Furthermore, the risk of bleeding may be increased by the concomitant use of anticoagulants and antiplatelet drugs, which is very common in clinical practice [33,34,35].

## 7. Conclusions

GIB is a common cause of hospitalisation and often requires reference centres and a multidisciplinary approach that includes emergency physicians; internal medicine specialists; surgeons; gastroenterologists; diagnostic and interventional radiologists. While many patients with GIB resolve spontaneously or may benefit from medical and endoscopic therapies, in other cases the use of imaging beyond endoscopy appears to be decisive. In this context, among the imaging methods, CTA is the gold standard in the timely and accurate diagnosis of GIBs, with high sensitivity and clinical efficacy in guiding the choice of the correct therapeutic strategy. The dual-energy acquisition techniques also allow for the optimisation of contrast resolution, facilitating the identification of haemorrhagic foci using lower doses of contrast medium and lower radiation doses applied to the patient being examined.

## Figures and Tables

**Figure 21:**
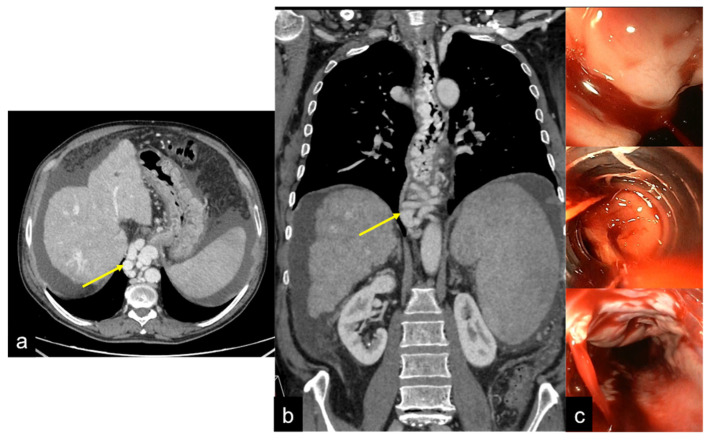
Axial (**a**) and coronal (**b**) images of CTA multiphasic study with endoscopic corresponding images (**c**). In CT, the oesophageal varices in portal hypertension are visualised as tubular structures with an endoluminal protrusion in the oesophageal wall ((**a**,**b**) arrow); the endoscopic evaluation reveals signs of active bleeding (**c**).

**Figure 22:**
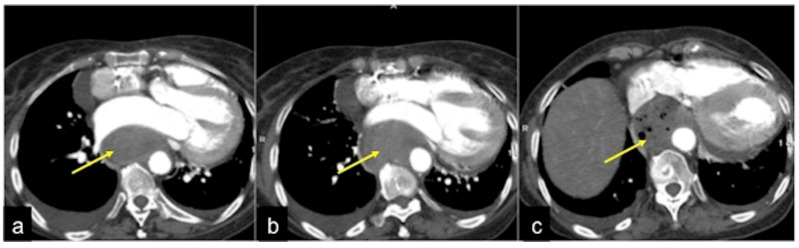
CTA images of Mallory–Wess lesion in the arterial phase. Oedematous thickening oesophageal wall ((**a**,**b**) arrow) with small gas bubbles around the laceration site (**c**, arrow).

**Figure 23:**
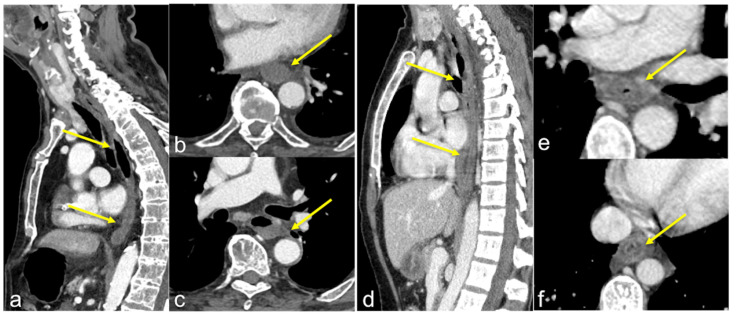
Two different patients with consequences of caustic ingestion on the oesophagus. Enhanced CTA scan in the portal venous phase in sagittal (**a**,**d**) and axial (**b**,**c**,**e**,**f**) views. Note, in the first case (**a**–**c**) the extensive oesophageal necrosis (**a**–**c**, arrows) with a thin and unenhanced wall, in the second case (**d**–**f**) the oesophageal thickening with a stratified wall (**d**–**f**, arrows).

**Figure 24:**
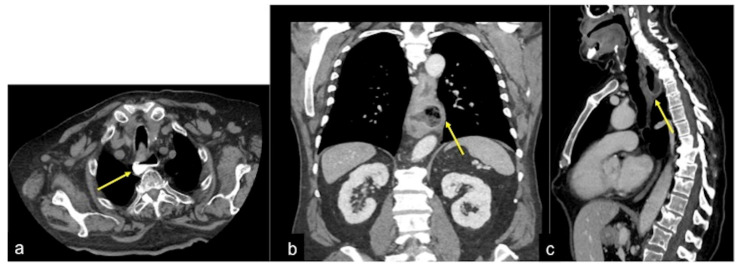
Oesophageal diverticula. Axial (**a**), coronal (**b**) and sagittal (**c**) images of the CTA multiphasic study show a wall protrusion in the middle oesophagus with mucosal hyperaemia as a possible site of bleeding (**a**–**c**, arrow).

**Figure 25:**
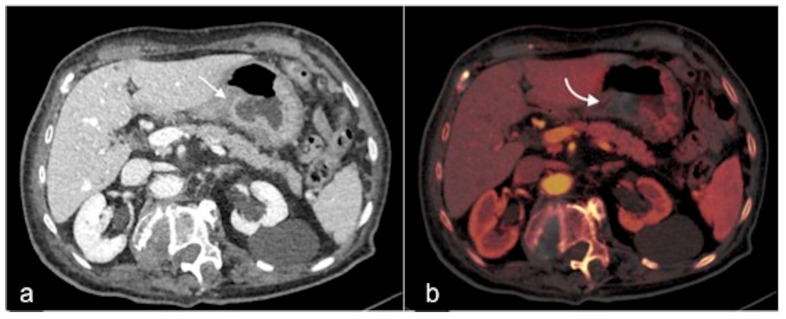
DECTA of peptic ulcer of the stomach. The arterial phase (**a**) and relative iodinated map (**b**) show large ulceration in the gastric body (arrow (**a**)) with hyperaemic wall area; no active bleeding signs are demonstrated in the iodinated map (curved arrow (**b**)).

**Figure 26:**
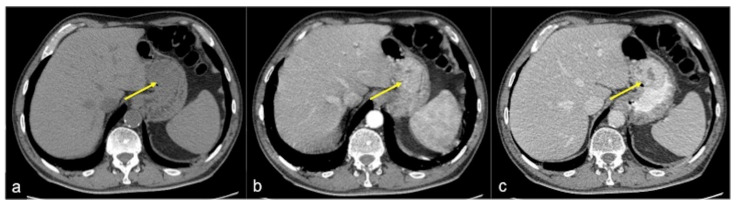
CTA multiphasic study of gastric GIST. In the pre-contrast (**a**), arterial (**b**) and venous (**c**) phases a large vascularised lesion in the gastric body with massive haemorrhagic lumen inundation is visible (**a**–**c**, arrow).

**Figure 27:**
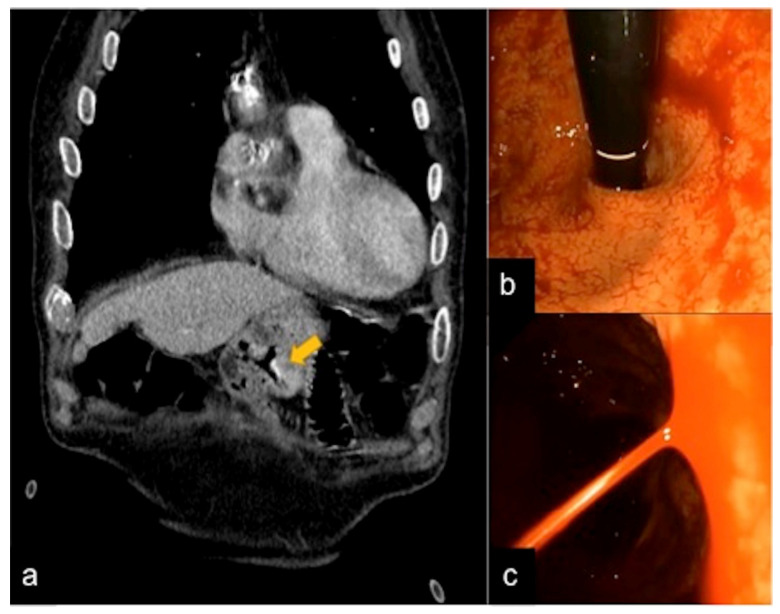
CTA multiphasic study of a gastric Dieulafoy lesion. In the arterial phase (**a**) and relative endoscopic evaluation (**b**,**c**), a vascular sub-mucosal lesion is identified (**a**, arrow).

**Figure 28:**
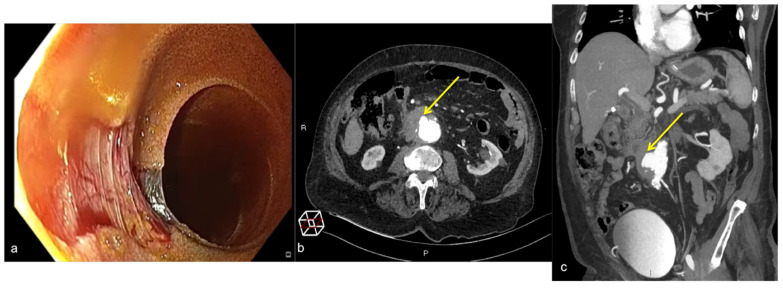
Hematochezia and abdominal pain with pulsatile mass. Fresh blood on colonoscopy without bleeding cause. Upper endoscopy (**a**) shows active pulsatile bleeding at the third duodenal portion. Axial CTA artery phase (**b**) and coronal MIP artery phase reconstruction (**c**) show a large outpouching from the right anterolateral wall of the abdominal aorta at the level of the third duodenal portion with loss of interface fat plane ((**b**,**c**) long arrows), The outpouching contains mural thrombosis. No air bubble is depicted in the aortic lumen or wall. No contrast extravasation into the duodenum is seen.

**Figure 29:**
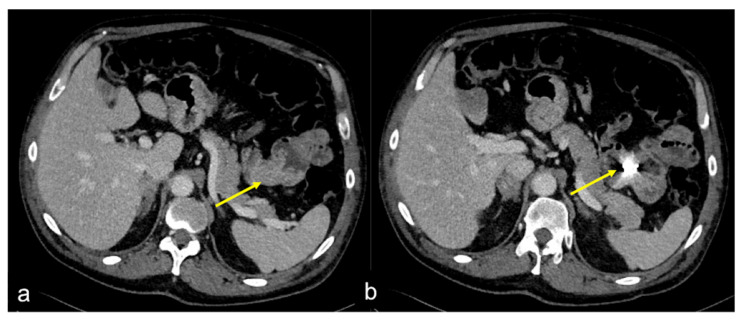
CTA study in venous phase (**a**) of jejunum adenocarcinoma shows a substenosing solid mass in the lumen of the jejunum (arrow); the successive check after endoscopic haemostasis (**b**, arrow).

**Figure 30:**
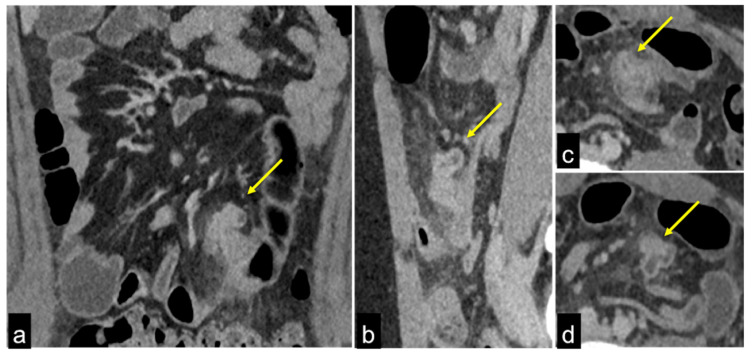
Fifteen-year-old patient complaining of abdominal pain and anaemia. Enhanced CTA scan in portal venous phase in coronal (**a**), sagittal (**b**), axial (**c**,**d**) views. In the mesogastric region, there is a diverticular ileal structure (**a**–**d**, arrows) with increased wall enhancement at CTA venous phase, associated with adjacent fat stranding. Findings are consistent with Meckel diverticulitis.

**Figure 31:**
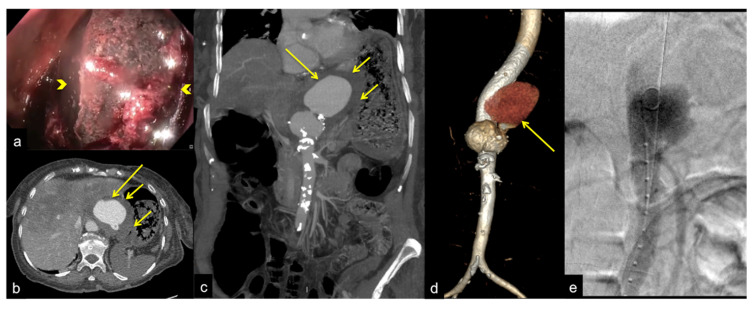
Abdominal pain and anaemia. Gastric endoscopy (**a**) shows an extrinsic bulging mass in the gastric corpus with a large adherent blood clot ((**a**) arrowheads). Axial CTA artery phase (**b**) and coronal MIP artery phase reconstruction (**c**) show a contained rupture of a thoraco-abdominal aortic aneurysm ((**b**,**c**) long arrows) with periaortic haematoma compressing the posterior gastric wall with loss of interface fat plane ((**b**,**c**) short arrows). The VR-3D artery phase image reconstruction of the aortic aneurism (**d**, arrow) better defines the extension of the aneurysm sac ((**d**) long arrow) for endovascular operative planning. Angiography endovascular aneurysm repair procedure (**e**).

**Figure 32:**
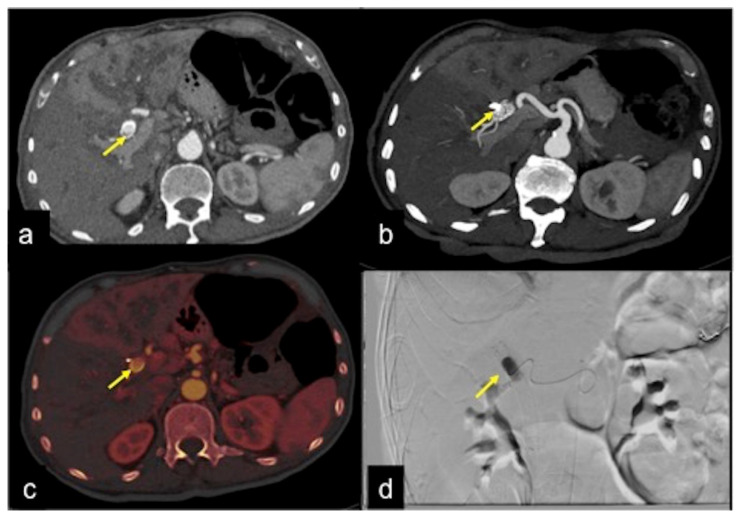
DECTA study of iatrogenic embolism. On arterial phase (**a**) with MIP reconstruction (**b**) and relative iodinated map (**c**) a pseudoaneurysm in the lumen of the biliary stent is detected ((**a**–**c**) arrow). The angiography (**d**) identifies the pseudoaneurysm for exclusion treatment (arrow).

**Figure 33:**
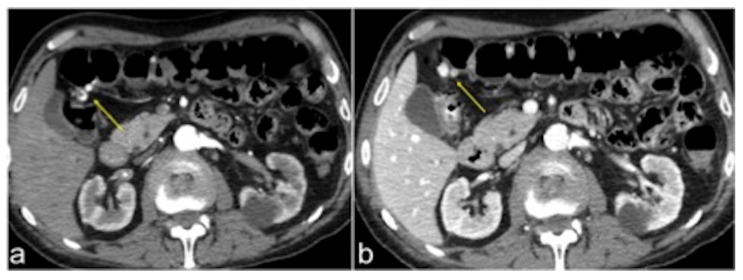
CTA multiphasic study in the arterial (**a**) and venous (**b**) phases of diverticular bleeding; a haemorrhagic spot in a diverticular sac of the colon at hepatic flexure is visible in the arterial phase (**a**, arrow) with increase and intensification in the venous phase (**b**, arrow).

**Figure 34:**
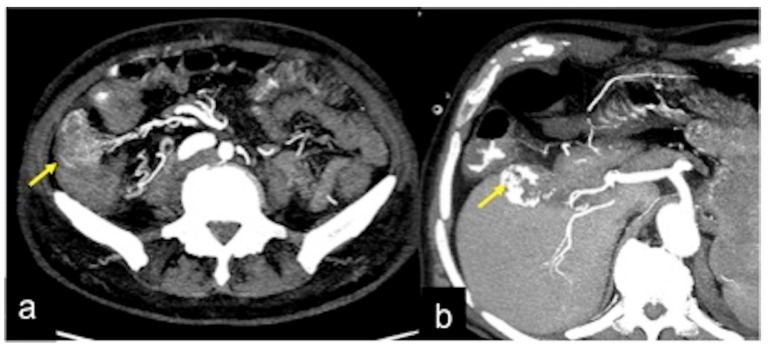
CTA multiphasic study of hepatic colon flexure angiodysplasia. In the arterial phase (**a**) and MIP reconstruction (**b**), it is possible to detect a haemorrhagic focus contiguous to a series of tortuous blood vessels in the colon wall (**a**,**b**, arrow).

**Figure 35:**
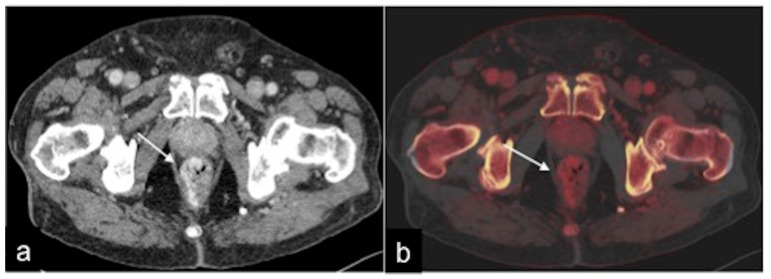
DECTA study of rectal varices. In the venous phase (**a**) and relative iodinated map (**b**), it is possible to identify venous ectasic structures in the rectal wall (**a**,**b**, arrow).

**Figure 36:**
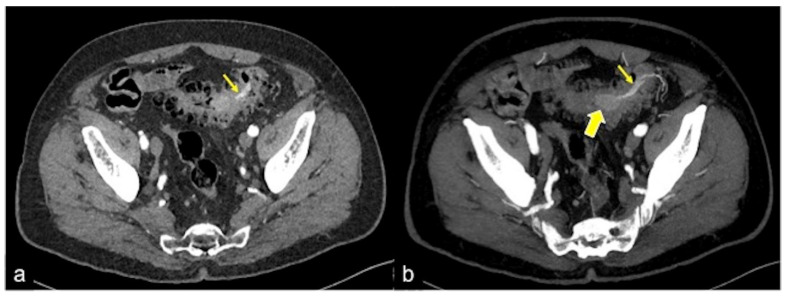
CTA multiphasic study of pedunculated polyp of the sigma. In the arterial phase (**a**) and relative MIP (**b**), it is possible to define an arterial vascular axis (**a**,**b**, short arrow) supporting a large polyploid formation (**b**, large arrow).

**Figure 37:**
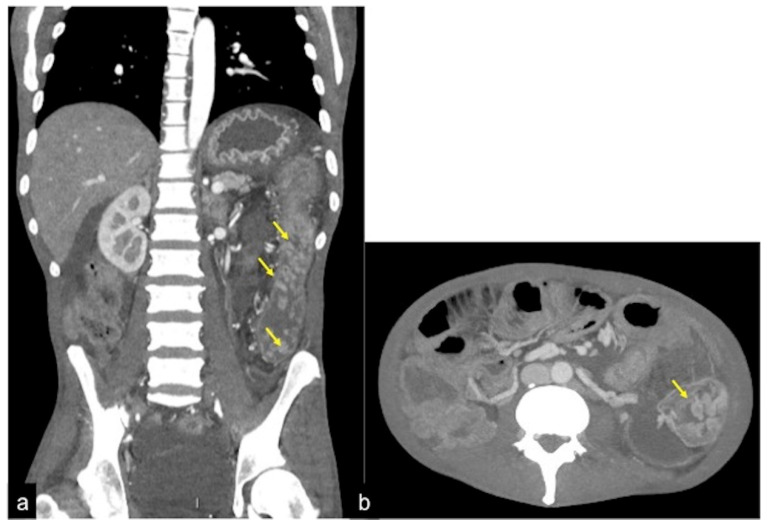
CTA arterial phase (**a**) and relative MIP reconstruction (**b**) of polyposis of the descending colon (**a**,**b**, arrows).

**Figure 38:**
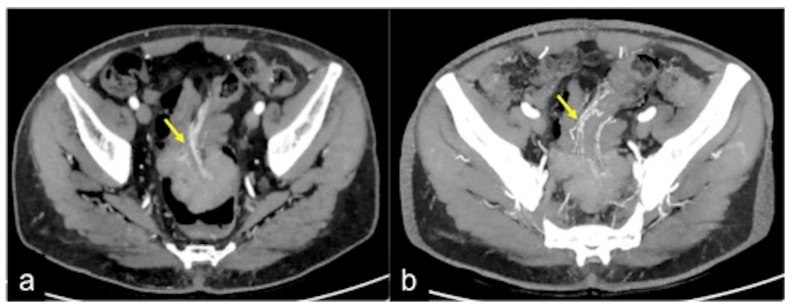
CTA multiphasic study in the arterial phase (**a**) and MIP reconstruction (**b**); an invaginated polyp in the sigmoid colon with arterial vascular support is visible (**a**,**b**, arrow).

**Figure 39:**
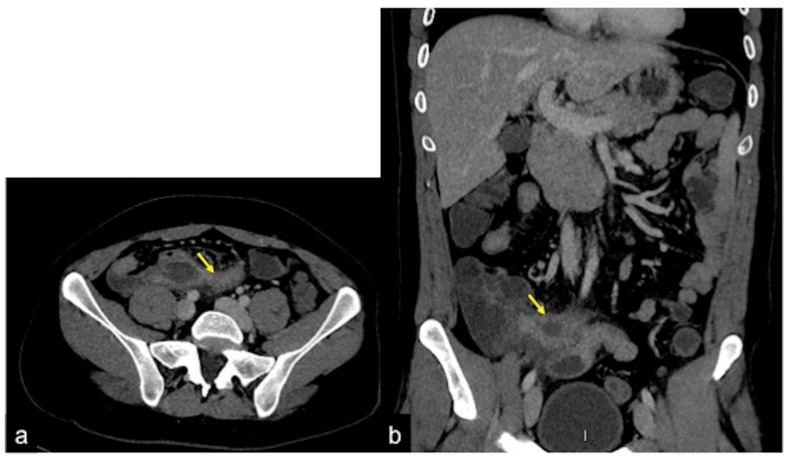
CTA axial (**a**) and coronal (**b**) images in the venous phase show inflammatory stenosis of terminal ileum ((**a**) arrow) in Crohn’s disease complicated by an abscess ((**b**) arrow).

**Figure 40:**
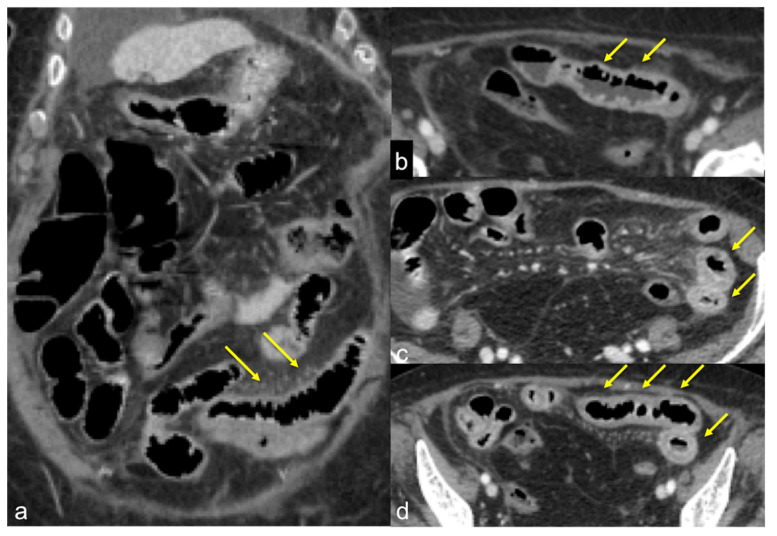
Reactivation of Crohn’s ileitis. Enhanced CTA scan in portal venous phase in coronal (**a**) and axial (**b**–**d**_**b;c;d;d;e;f;d;e;f;a;b;c;a;b;c;d**_) views. Note the ileal wall thickening with segmental irregularity and hyperaemia of the mucosa (**a**–**d**, arrows).

**Figure 41:**
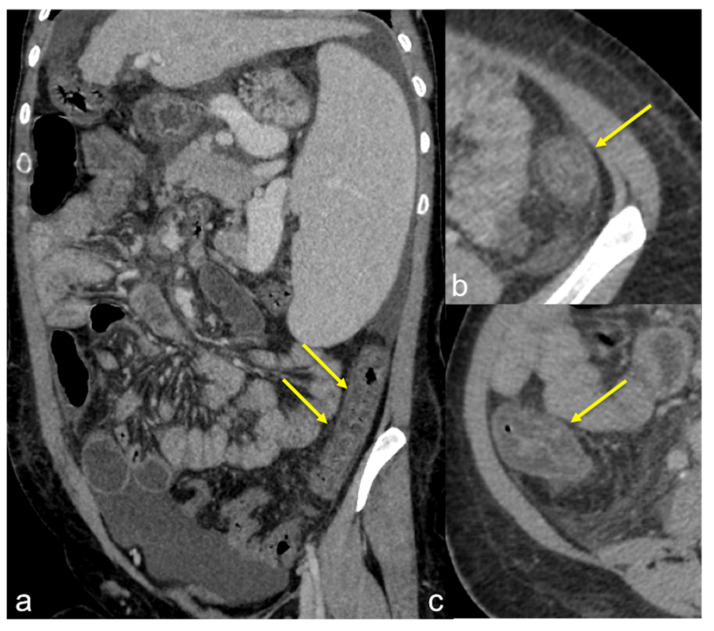
Ischaemic colitis in a cirrhotic patient. Enhanced CTA scan in portal venous phase in coronal-oblique (**a**) and axial (**b**,**c**) views. Note the typical colonic involvement (**a**–**c**_**a;b;c;b;c;d;d;e;f;d;e;f;a;b;c;a;b;c;d**_) arrows) with stratified wall thickening with submucosal oedema, mucosal hyperaemia and adjacent mesenteric stranding.

**Table 1:** Clinical terminology and glossary of clinical signs in gastrointestinal bleeding.

Clinical Terminology by Type of Gastrointestinal Bleeding	
UGIB	Upper gastrointestinal haemorrhage—proximal to the Treitz ligament (oesophagus, stomach and duodenum).
LGIB	Lower GI haemorrhage—distal to the Treitz ligament (colon and rectum).
SBB/MGIB	Middle gastrointestinal haemorrhage—distal to the ampulla of Vater and proximal to the ileocaecal valve (small intestine).
Suspected SBB	Gastrointestinal haemorrhage in which no source of bleeding is identified after performing both upper and lower endoscopy.
Obscure GIB	Gastrointestinal haemorrhage in which no source of bleeding is identified after the entire gastrointestinal tract has been fully evaluated with both endoscopic and advanced *imaging* techniques. Obscure gastrointestinal bleeding can be overt or occult depending on whether evident gastrointestinal bleeding is clinically present.
Overt GIB	Visible gastrointestinal bleeding such as haematemesis, haematochezia or melaena. The definition of overt haemorrhage is preferable to that of acute haemorrhage because the latter only defines the onset of symptoms and not the visibility of the bleeding itself. In addition, patients with overt GIB may present acutely, but also intermittently or for a prolonged period.
Occult GIB	Gastrointestinal bleeding that is not clinically visible. Patients with occult gastrointestinal bleeding have a positive faecal occult blood test or iron deficiency anaemia with no apparent cause.
Massive GIB	Gastrointestinal bleeding associated with hemodynamic instability (e.g., hypotension with systolic blood pressure <90 mmHg, tachycardia, symptoms of pre-shock or shock) or bleeding that requires transfusion of more than 4 units of packed red blood cells in 24 h.
Haematemesis	This term refers to the vomiting of blood and is indicative of bleeding from the oesophagus, stomach or duodenum. Haematemesis includes the vomiting of bright red blood, which suggests recent or current blood loss, and vomiting of coffee-like material (coffee grounds vomiting) which suggests the bleeding stopped some time ago.
Melaena	It consists of black liquid stools resulting from the breakdown of blood into haematin or other haemoglobin components by intestinal bacteria. Melaena is the manifestation of bleeding that originates from the upper GI tract, the small intestine, or the proximal portion of the large intestine. It generally occurs when 50 to 100 mL or more of blood (usually from the upper GI tract) is present in the alimentary canal, with characteristic stool passed a few hours after the bleeding event.
Haematochezia	It consists of bright red blood evacuated from the rectum and suggests active bleeding from the proximal GI tract, small intestine, distal colon or anorectal area.
Rectorrhagia	It consists of severe bleeding from the distal GI tract.

**Table 2:** Recommendations, advantages and limitations of the use of the various endoscopic and *imaging* techniques.

	Recommendations	Advantages	Disadvantages
**Endoscopy**	Initial procedure for the diagnosis and treatment of UGIB and LIGB in haemodynamically stable patients.	Locates the source of bleeding and allows for haemostatic treatment in most patients. Tissue samples can be taken in the event of suspected malignancy.	It may not be universally available. Limitations in terms of the visualisation of the whole small intestine. Diagnostic exploration limited by the presence of abundant active bleeding, food residues and faeces.
**Enteroscopy and Videocapsule Endoscopy (VDE)**	Possible diagnostic use in the detection of focal SBB/MGIB and LGIB bleeds in haemodynamically stable patients. Endoscopic procedures, such as single or double balloon enteroscopy or retrograde ileoscopy may be indicated to define the diagnosis and treatment if the source of bleeding was found with the VDC.	Visualises the small intestine mucosa directly with the ability to identify a potential source of active bleeding.	Enteroscopy: an invasive procedure that requires sedation. Risk of perforation. Need for specialised staff and level II centres. It is not used in UGIB. VDC: long duration of examination and analysis time of acquired images (>8 h); furthermore, it does not allow for therapeutic haemostasis. Need for specialised staff and level II centres. It is not used in UGIB.
**Scintigraphy**	Possible diagnostic use in the search for focal SBB/MGIB and LGIB in haemodynamically stable patients.	It can detect low rates of arterial or venous bleeding, including intermittent. Non-invasive. No bowel preparation required.	Often, it does not allow for the exact identification of the bleeding site. High radiation dose applied. It requires time and specialised staff that are not available in urgent contexts. It is not used in UGIB.
**Angiography**	Possible diagnostic use in the screening for focal SBB/MGIB and LGIB in haemodynamically stable patients. Recurrent/continuous bleeding after colonoscopic treatment for LGIB. For UGIB, patients with acute bleeding with negative endoscopy or in whom endoscopy could not find the source (especially haemodynamically unstable patients). Possible therapeutic use for focal UGIB, MGIB and LGIB that cannot be treated endoscopically.	It can identify and treat gastrointestinal bleeding, if detected, also allowing for the selective exclusion of small vessels. High spatial resolution.	It requires a high bleeding rate for detection of the bleeding source. Invasive and time-consuming procedure for diagnostic purposes. High radiation dose applied. Requires the use of contrast media. Risk of bowel wall ischaemia [6,7,8]. Need for specialised staff and level II centres [9,10,11].
**Magnetic Resonance Imaging (MRI)**	Possible use for diagnosis and follow-up of IBD. Not indicated for the detection of active GI bleeding. Non-routine use in occult bleeding and obscure SBB/MGIB in haemodynamically stable patients.	High contrast and spatial resolution. Possibility of using ultra-fast sequences to examine intestinal motility (cine-MRI). No ionising radiation.	Not widely available. Long procedure. Need for proper preparation. Use of contrast media. Not therapeutic. It is not used in UGIB.
**CT**	The technique of choice for all SBB/MGIB and LGIB with active bleeding in hemodynamically stable (or stabilised) patients; UGIB with negative endoscopy, or endoscopy unable to identify the source (comparable to angiography).	Widely available. Quick identification of the bleeding source with precise anatomical localisation. Containment of the radiation dose with advanced technology (*dual energy*).	It may be less sensitive than radionuclide *imaging*. It can underestimate intermittent bleeding. Not therapeutic. Radiation dose. Requires the use of contrast medium.

**Table 3:** CTA protocol for overt GIB and optional CTE protocol for occult GIB or SBB.

	**Overt GIB—CTA Protocol**	**Occult GIB or SBB—CTE Protocol**
IV administration of contrast medium	80–130 mL of high-concentration iodinated contrast medium (370–400 mgI/mL)	80–130 mL of high-concentration iodinated contrast medium (370–400 mgI/mL)
Speed of administration	The highest possible flow (3.5–4 mL/s) through an 18G cannula	The highest possible flow (3.5–4 mL/s) through an 18G cannula
Normal saline	40 mL of high-flow normal saline	40 mL of high-flow normal saline
Oral administration of contrast medium	Not recommended	1350–1500 mL of fractionated neutral oral contrast agent starting about 1 h prior to the examination
Scanned area	From the diaphragm to the pubic symphysis (possible extension to the chest)	From the diaphragm to the pubic symphysis
Phases of acquisition	Multiphase CT technique: Without contrast or virtual no contrast Arterial phase (*bolus tracking* technique) Venous phase (70–90 s after injection) Optional late phase (5 min after injection)	Multiphase CT technique: Without contrast or virtual no contrast Late arterial phase (10 s after bolus trigger) Enteric phase (50 s after injection) Late venous phase (90 s after injection) Alternative technique: split bolus protocols may be adopted
Post-processing	2.5–3 mm axial slices for each series (optional 1 mm axial) Coronal and sagittal reconstruction of 2.5–3 mm images (50% overlap) Optional maximum intensity projection and volumetric reconstruction	2.5–3 mm axial slices for each series (optional 1 mm axial) Coronal and sagittal reconstruction of 2.5–3 mm images (50% overlap) Optional maximum intensity projection and volumetric reconstruction
DECTA post-processing	40–60 keV (i.e., virtual monoenergetic), iodine density, virtual non-contrast and standard mixed series	40–60 keV (i.e., virtual monoenergetic), iodine density, virtual non-contrast and standard mixed series

**Table 4:** Pitfalls in the detection of acute gastrointestinal bleeding with CTA/DECTA.

**FALSE NEGATIVE**	
**Pitfall**	**Prevention Strategy**
Fluid-filled dilated bowel	No positive or negative contrast media administration (Figure 19)
Low flow rate of contrast media administration	Rigorous CTA/DECTA examination technique; post-processing reconstructions (Figure 11 and Figure 14)
Low-flow bleeding or reduced cardiac function/hypovolaemic or septic shock etc.	CTA/DECTA late venous phase; post-processing reconstruction (Figure 4 and Figure 9)
**FALSE POSITIVE**	
**Pitfall**	**Prevention Strategy**
Suture material, clips, foreign bodies	Unenhanced CTA scan; virtual unenhanced DECTA reconstruction; DECTA iodine map (Figure 2, Figure 7, Figure 8, Figure 16 and Figure 19)
Retained contrast media within the lumen after previous contrast media administration	Unenhanced CTA scan; virtual unenhanced DECTA reconstruction (Figure 19)
Cone-beam artefacts	Rigorous CTA/DECTA examination technique; post-processing reconstructions (Figure 19)

## Data Availability

Not applicable.
